# TIMP1 Overexpression in Ovarian Cancer Spheroids: Implications for Prognosis, Resistance, and Metastatic Potential

**DOI:** 10.3390/cancers17101605

**Published:** 2025-05-09

**Authors:** Andrea Jemma, Alessandra Ardizzoia, Chiara Villa, Sara Bonomo, Mario Mauri, Carla Reale, Concetta Ambrosino, Massimiliano Cadamuro, Marialuisa Lavitrano, Donatella Conconi

**Affiliations:** 1 School of Medicine and Surgery, University of Milano-Bicocca, Via Cadore 48, 20900 Monza, Italy; alessandra.ardizzoia@ifom.eu (A.A.); chiara.villa@unimib.it (C.V.); sara.bonomo@unimib.it (S.B.); mario.mauri@unimib.it (M.M.); massimiliano.cadamuro@unimib.it (M.C.); marialuisa.lavitrano@unimib.it (M.L.); 2 IFOM, The AIRC Institute for Molecular Oncology, Via Adamello 16, 20139 Milano, Italy; 3 Biogem Scarl, Institute of Molecular Biology and Genetics Research, Via Camporeale, 83031 Ariano Irpino, Italy; carla.reale@biogem.it (C.R.); coambros@unisannio.it (C.A.); 4 Department of Science and Technology, University of Sannio, Via de Sanctis, 82100 Benevento, Italy; 5 Department of Biology, University of Naples Federico II, Via Cinthia 26, 80126 Napoli, Italy

**Keywords:** ovarian cancer, TIMP1, cancer stem cells, prognostic biomarker

## Abstract

**Background:** High dissemination potential and resistance to standard therapy significantly contribute to high mortality associated with ovarian cancer. Cancer stem cells (CSCs) drive tumor progression, metastasis, and recurrence after treatments’ failure. Here, we provide the first evidence of TIMP1 overexpression in ovarian CSCs, suggesting its potential role as a prognostic biomarker. **Methods:** Different ovarian cancer cell models were used to explore the potential link between TIMP1 and stem-like phenotypes. Experiments included spheroid formation, drug treatments, gene expression, functional assays, and zebrafish xenograft models to assess cell behavior and molecular changes. **Results:** TIMP1 was overexpressed in CSCs, and its expression was also upregulated in chemoresistant and anoikis-resistant cells. Our database analysis revealed a correlation between TIMP1 expression levels and poor patient prognosis. Overexpression of TIMP1 in ovarian cancer cell lines was able to recapitulate several features of the ovarian cancer stem cell phenotype, including treatment resistance, expression of stem cell markers, and anoikis resistance. TIMP1-overexpressing cells also exhibited enhanced migration potential in vitro and increased metastatic potential in vivo. Moreover, TIMP1 overexpression significantly altered the transcriptome landscape of cells, highlighting its role in modulating critical pathways associated with cell migration and inflammation. **Conclusions:** This study identifies the pivotal role of TIMP1 in ovarian CSCs and its contribution to therapy resistance, recurrence, and metastasis.

## Simple Summary

Ovarian cancer has high mortality due to its dissemination potential and resistance to therapy, with cancer stem cells (CSCs) playing a key role. This study provides the first evidence of TIMP1 overexpression in ovarian CSCs and its correlation with poor patient prognosis, suggesting its potential as a prognostic biomarker. Overexpression of TIMP1 in ovarian cancer cell lines recapitulated CSC features, including treatment and anoikis resistance, stem cell marker expression, and enhanced migration. TIMP1-overexpressing cells also showed increased metastatic potential in vivo and altered gene expression, particularly in pathways related to migration. This study highlights TIMP1’s role in ovarian CSCs, contributing to therapy resistance, recurrence, and metastasis.

## 1. Introduction

Ovarian cancer (OC) is the seventh most common malignancy worldwide and the eighth most common cause of cancer death in the female population [1]. Due to its high heterogeneity, it is represented by a diverse group of neoplasms with different molecular signatures and prognoses [2]. Epithelial OC accounts for 90% of ovarian malignancies and includes various histotypes, with high-grade serous carcinoma (HGSC) being the most prevalent and lethal, responsible for 70–80% of OC-related mortality [3]. HGSC is usually diagnosed at an advanced stage, and patients often develop chemoresistance after an initial response to first-line therapy, which includes surgery followed by platinum/taxane-based chemotherapy [4,5]. This results in a high recurrence rate (75–80%) over three years [6]. The lack of prognostic biomarkers makes it challenging to predict patients’ response to first-line therapy and their prognosis [7]. HGSC is also characterized by high dissemination potential, and tumor cells typically spread throughout the abdomen or the abdominal cavity [8]. HGSC dissemination is strongly related to malignant ascites, which is found in one-third of patients at the time of diagnosis and in nearly all patients following cancer relapse [9]. Additionally, spheroids in ascitic fluid contribute to both metastasis and the acquisition of chemoresistance, exhibiting stem-like properties, including self-renewal, increased expression of cancer stemness genes, higher invasiveness, migration potential, and enhanced chemoresistance [10,11]. Nowadays, several studies have highlighted the clinical relevance of cancer stem cells (CSCs) in the progression, metastasis, and relapse of OC, making them one of the main putative targets for preventing tumor recurrences and overcoming chemoresistance [12].

Tissue Inhibitor of Metalloproteinases 1 (TIMP1) is a member of the Tissue Inhibitors of Metalloproteinases family, involved in maintaining, remodeling, and regulating the turnover of the extracellular matrix during both physiological and pathological processes. Given its functions, TIMP1 has been linked to reduced cell invasion and metastasis; however, its overexpression has also been associated with increased cell growth and inhibition of apoptosis, contributing to cancer progression [13]. In this manuscript, we report for the first time the overexpression of TIMP1 in OC stem cells, suggesting its involvement in regulating stemness potential. TIMP1 expression correlates with reduced sensitivity to standard therapy, acquired anchorage-independent growth ability, and powered migration potential. Our data indicate that TIMP1 may serve as a novel putative prognostic biomarker in HGSC.

## 2. Materials and Methods

### 2.1. Cell Lines

In this study, the human ovarian cancer cell lines Ovcar5 and Ovcar8 were used to investigate the role of cancer stem cell-like properties in ovarian cancer progression and chemoresistance. Ovcar5 and Ovcar8 were purchased from Sigma-Aldrich (St. Louis, MO, USA) and grown in RPMI 1640 containing 10% Fetal Bovine Serum (FBS). 1% Penicillin/Streptomycin was added to all culture media. Cell lines were grown adherent to plastic in T25 or T75 flasks and kept at 37 °C with 5% CO_2_ in a humidified atmosphere. Media, sera, and supplements were all from EuroClone (Milano, Italy).

Authentication of these cell lines was performed through periodical ploidy evaluation and an array-CGH analysis as previously reported [6]. All experiments were conducted with mycoplasma-free cells, with regular testing for mycoplasma contamination using MycoAlert^®^ Mycoplasma Detection Kit (Lonza, Basel, Switzerland). These steps ensure that the cell lines used in this study are authentic and free from contaminants, thus supporting the reproducibility and reliability of our findings.

### 2.2. Ovarian Cancer Spheroids

Ovarian cancer spheroids were generated by an anchorage-independent culture system as previously described [6]. Briefly, the ovarian cancer cell lines Ovcar5 and Ovcar8 (10^6^/mL) were suspended in Dulbecco’s Modified Eagle’s Medium (DMEM) F-12 with 1% Penicillin/Streptomycin (Euroclone, Milano, Italy), supplemented with B27 (2.5 mL/L, Life Technologies, Carlsbad, CA, USA), epidermal growth factor (EGF, 20 ng/mL, Miltenyi Biotec, Singapore), and basic fibroblast growth factor (bFGF, 10 ng/mL, Miltenyi Biotec, Singapore) to facilitate spheroid formation. To validate the models, the ability to form spheroids from a single cell for at least five passages and the expression of ovarian stemness markers (ALDHA1, CD44, ABCG2, and NANOG) were evaluated [14]. The ovarian cancer spheroids were maintained in a humidified atmosphere at 5% CO_2_ and 37 °C.

### 2.3. Cell Treatments and Reagents

For in vitro studies, paclitaxel and carboplatin (Selleckchem, Houston, TX, USA) were reconstituted at a final concentration of 10 mM in DMSO and water, respectively, and stored in aliquots at −80 °C. Recombinant Human TIMP-1 Protein, CF (970-TM) and Human TIMP-1 Antibody (AF970) were both purchased by Bio-techne (Minneapolis, MN, USA) and reconstituted in deionized water and PBS, respectively, according to product instructions.

### 2.4. Cell Viability Assays

For all ovarian cancer models, 1.5–2 × 10^4^ cells/well were seeded in triplicate in a 96-well plate. After 24 h (60–70% confluence), the medium was replaced with fresh medium alone or containing drugs. After 72 h of treatment, cell viability was evaluated by an MTT assay. The MTT (3-(4,5-Dimethylthiazol-2-yl)-2,5-diphenyltetrazolium bromide) solution (Sigma-Aldrich, St. Louis, MO, USA) was added to each well (0.5 mg/mL), incubated for 3 h at 37 °C, and then, formazan was solubilized in absolute ethanol. The absorbance was measured spectrophotometrically at 570 nm wavelength using an automated microplate reader (TECAN Infinite M200 PRO, Tecan, Männedorf, Switzerland). The values on the x-axis represent log-transformed concentrations of paclitaxel and carboplatin. Specifically, paclitaxel concentrations are expressed in nanomolar (nM) and carboplatin concentrations in micromolar (µM). The IC50 values were calculated based on these log-transformed concentrations using GraphPad software (Prism version 5). All the data are expressed as the mean of three different experiments.

### 2.5. Ovarian Cancer Chemoresistant Cells

Ovcar5 and Ovcar8 cells were treated with increasing doses of paclitaxel and carboplatin for 48 h; this was followed by a recovery period in drug-free medium until they reached 70% confluence. The cells that survived were then exposed to concentrations up to twice their respective half-maximal Inhibitory Concentration (IC50) for both drugs. The acquisition of chemoresistance was confirmed using MTT (3-(4,5-Dimethylthiazol-2-yl)-2,5-diphenyltetrazolium bromide) assay. As chemoresistance was confirmed, cells were maintained in RPMI 1640 supplemented with 10% FBS and 1% Penicillin/Streptomycin (Euroclone, Milano, Italy), along with paclitaxel and carboplatin at concentrations equal to the IC50 values for each cell line, in a humidified environment at 5% CO_2_ and 37 °C.

### 2.6. Ovarian Anoikis-Resistant Cells

Anoikis-resistant cells were generated by alternating sequential adherent and non-adherent cycles of culturing as described by Hasseri Halim et al. [15]. Briefly, Ovcar5 and Ovcar8 cells were seeded onto cell-repellent surface 6-well culture plates for 72 h, in RPMI 1640 supplemented with 10% FBS and 1% Penicillin/Streptomycin (Euroclone, Milano, Italy). After this incubation period, the cells were collected and seeded under adherent conditions to allow recovery. Subsequently, the expanded cultures were collected and seeded again in cell-repellent surface 6-well culture plates (Greiner Bio-One, Kremsmünster, Austria) for 72 h. Each cycle was repeated 5 times, always in RPMI 1640 supplemented with 10% FBS and 1% Penicillin/Streptomycin medium.

### 2.7. Production of TIMP1 Vector

F2A system-based dicistronic vector for the expression of human TIMP1 was obtained following a strategy similar to that previously reported by Ryan and Drew [16]. To facilitate the detection of the transfected cells, the vector also encoded the e-GFP (enhanced green fluorescent protein) as a valuable reporter molecule. Briefly, the coding sequence (CDS) of TIMP1 (NCBI: NM_003254.3) was PCR-amplified with primers introducing the XhoI site both at the 5′ and 3′ ends of the sequence and cloned into the XhoI-linearized pCX-eGFP-F2A plasmid acceptor downstream of the F2A sequence, as previously described [17]. The final construct, named pCX-eGFP-F2A-TIMP1 (TIMP1 vector and empty vector as negative control), was confirmed for the correct orientation by restriction enzyme analysis. To exclude non-homologous recombination events or mutations arising from the procedure, the nucleotide sequence of the CDS was confirmed by sequencing analyses performed on both strands using an automated ABI-3130 DNA sequencer (Applied Biosystems, Foster City, CA, USA). All plasmids were purified using the QIAGEN Plasmid Maxiprep kit (QIAGEN, Hilden, Germany) according to the manufacturer’s protocol and resuspended in water.

### 2.8. Ovarian Cells Overexpressing TIMP1

For TIMP1-overexpressing ovarian cancer cells, Ovcar8 and Ovcar5 were stably transfected with the TIMP1 vector or the negative control (empty vector) using the Lipofectamine^®^ 3000 reagent (Invitrogen, Carlsbad, CA, USA) according to the manufacturer’s instructions. Briefly, 4 × 10^5^ cells per well were seeded in 6-well plates, in RPMI 1640 with 10% FBS without Penicillin/Streptomycin. The transfection complex, composed of 2500 ng of the transfection vector, 3.75 µL of the Lipofectamine™ 3000 transfection reagent, and the P3000™ reagent, all suspended in Opti-MEM^®^ I medium (Gibco, Grand Island, NY, USA), was added to each well and incubated for 24/48 h. For stable transfection, the transfected ovarian cancer cells were selected in fresh medium in the presence of 700 µg/µL of G418 (Euroclone, Milano, Italy) for 2 weeks.

### 2.9. RNA Extraction and Real-Time PCR

Total RNA from all ovarian cancer models was isolated by RNeasy Mini Kit according to the manufacturer’s instructions (QIAGEN, Hilden, Germany). RNA quantity and quality were evaluated with a Nanodrop ND2000 spectrophotometer (Thermo Fisher Scientific, Waltham, MA, USA). After measuring the concentrations, total RNA was reverse-transcribed using the High-Capacity cDNA Reverse Transcription Kit (Thermo Fisher Scientific, Waltham, MA, USA). For each sample, 1 µg of RNA was reverse-transcribed. Real-time PCR was performed using TaqMan Gene Expression Master Mix (Applied Biosystems, Waltham, MA, USA), and the plates were analyzed by thermocycler StepOnePlus Real-time PCR System (Applied Biosystems, Waltham, MA, USA). All TaqMan probes, ALDH1 (Hs00946916_m1), ABCG2 (Hs01053709_m1), CD44 (Hs01075861_m1) and NANOG (Hs04260366_g1), and TIMP1 (Hs99999139_m1), were purchased from Life Technologies (Waltham, MA, USA), and GAPDH (Hs99999905_m1) was used as the housekeeping gene. All experiments were performed in triplicate, and the results are expressed as the mean of at least three experiments.

### 2.10. Bulk RNA Sequencing

Total RNA from ovarian cancer models was isolated using the RNeasy Mini Kit according to the manufacturer’s instructions, as previously described (QIAGEN, Hilden, Germany). RNA quantity and purity were evaluated with a Nanodrop ND2000 spectrophotometer (Thermo Fisher Scientific, Waltham, MA, USA). At least 1.5 μg of total RNA was used as input material for RNA sample library preparation. Sample quality control, RNA-Seq, data collection, and data analysis were performed by Novogene Co., Ltd. (Beijing, China) using an Illumina platform. Differential expression analysis between two experimental conditions (three biological replicates per condition) was performed using the DESeq2 package in R (v1.20.0). *p*-values were adjusted using the Benjamini–Hochberg method to control the false discovery rate (FDR). Genes with an adjusted *p*-value < 0.05 were considered differentially expressed (DEGs). Subsequent pathway analysis included Gene Ontology (GO) enrichment analysis, and Kyoto Encyclopedia of Genes and Genomes (KEGG) enrichment analysis was conducted using the Cluster Profiler R package (Novogene, Beijing, China).

### 2.11. Western Blot

Cell lines, ovarian cancer spheroids, chemoresistant cells, anoikis-resistant cells, and TIMP1-overexpressing cells were collected. All samples were lysed in an appropriate volume of RIPA buffer (Hepes 50 mM, NaCl 500 mM, DTT 1 mM, EDTA 1 mM, 0.1% NP-40, pH 7.5) completed with 2% of protease and phosphatase inhibitors, 0.2% of EGTA and EDTA, and 0.1% of DTT. A minimum of 20 μg of cell lysates were separated on Novex 14% Tris-Glycine gels (Invitrogen, Carlsbad, CA, USA), transferred onto a nitrocellulose membrane (iBlot2 NC Mini Stacks, Invitrogen) and incubated with the following antibodies: anti-TIMP1 (D10E6, rabbit), anti-Bax (D2E11, rabbit), and anti-Bcl-xL (54H6, rabbit), all purchased by Cell Signalling Technology (Danvers, MA, USA). In all experiments, GAPDH (Sigma Aldrich, St. Louis, MO, USA) was used as the housekeeping gene. All images were acquired by the G:BOX XT4 Chemiluminescence and Fluorescence Imaging System (Syngene, Cambridge, UK). Each image was then analyzed and quantified through ImageJ 1.48v software. For each sample, the experiment was repeated at least three times (File S1).

### 2.12. Enzyme-Linked Immunosorbent Assay (ELISA)

Ovarian cancer cell lines, spheroids, TIMP1-vector-transfected cells (TIMP1-overexpressing cells), and empty-vector-transfected (empty cells) cells were seeded in 6-well plates at a density of 4 × 10^5^ cells per well in their respective culture media. After 24 h, the media were replaced with serum-free medium and incubated for an additional 24 h. The conditioned media were then collected and stored at −80 °C. The cell culture supernatants were used to detect the content of secreted TIMP1 using ELISA kits according to the manufacturer’s instructions (Human TIMP-1 ELISA Kit, Invitrogen, Carlsbad, CA, USA). TIMP-1 levels in medium were expressed in picomoles per liter. Absorbance was measured at 450 nm on an automated microplate reader (TECAN Infinite M200 PRO, Tecan, Männedorf, Switzerland).

### 2.13. Wound Healing Assay

Ovarian cancer cell lines, TIMP1-vector-transfected (TIMP1-overexpressing cells) and empty-vector-transfected (empty cells) cells were seeded at a density of 10^6^ cells/well into a 6-well plate and cultured in complete medium, to obtain a single confluent monolayer. The cell layer was wounded by a sterile pipette tip, washed with PBS several times, to remove all cellular fragments, and reincubated with fresh medium alone or medium containing Human TIMP-1 Protein or medium containing Human TIMP-1 Protein (10 μg/mL) and Human TIMP-1 Antibody (50 μg/mL) for 24 h, in a humidified atmosphere at 37 °C with 5% CO_2_. All scratches were acquired at 0 and 24 h, using a bright-field microscope and Digital C-Mount Camera TP 1400 (Sony, Milano, Italy), and all images were analyzed through ImageJ 1.48v software. The results are expressed as percentage of wound closure. For each sample, the experiment was repeated at least three times.

### 2.14. Soft Agar

A 1.2% low-gelling-temperature agarose (GellyPhorLE, Euroclone, Milano, Italy) stock solution was prepared in RPMI 1640, boiled, and kept at 40 °C in a water bath until use. A 0.6% of agarose solution, obtained by diluting the stock solution in RPMI 1640 completed with 20% FBS and 2% of Penicillin/Streptomycin, was added to a 6-well plate to prevent cell adhesion to the culture plate. Following this, a total of 3 × 10^3^ of ovarian cancer cell lines, TIMP1-overexpressing cells, or empty cells resuspended in 0.3% agarose solution were then seeded in the plates and transferred into a humidified cell culture incubator at 37 °C with 5% CO_2_ to allow for colony formation. Agar solution (0.3%) was produced by diluting 0.6% agarose solution in RPMI 1640 completed with 20% FBS and 2% of Penicillin/Streptomycin. The plates were stained on day 15 using MTT solution (0.5 mg/mL, Sigma-Aldrich, St. Louis, MO, USA), and pictures were acquired by a bright-field microscope, using the Sony Digital C-Mount Camera TP 1400. All colonies’ images were analyzed through ImageJ software, in order to quantify the size and colony number for each sample.

### 2.15. Zebrafish Model

All models were generated by Biogem Scarl (Institute of Molecular Biology and Genetics Research, Ariano Irpino, Italy). Animal experiments were performed in accordance with the European Council Directive 2010/63/EU. The Tg(fli1:EGFP) zebrafish line, with green fluorescent vessels, was raised under standard conditions. The eggs were obtained from natural spawning and maintained in an incubator at 28 °C for 48 h in E3 medium. Before the injection, Ovcar5 cells from the three different experimental groups were labeled with red cell tracker CM-Dil (Thermo Fisher Scientific, Waltham, MA, USA) according to manufacturer’s instructions. Two-day-old embryos were dechorionated and anesthetized with 0.04% of tricaine (Merck Millipore, Darmstadt, Germany), and approximately 150 cells/embryo were injected in the perivitelline space of each animal. Zebrafish larvae were anesthetized (with 0.04% tricaine) and evaluated at 24 and 72 h post-injection (hpi) using a fluorescence stereo microscope. Images of the embryos were captured with a Leica DFC450C camera and were analyzed with ImageJ software (National Institutes of Health, Rockville, MD, USA).

### 2.16. Bioinformatic Analysis

Kaplan–Meier Plotter (https://kmplot.com/analysis/; last access on 19 April 2025) and GEPIA2 (http://gepia2.cancer-pku.cn/#index, accessed on 19 April 2025) web servers were used to analyze the potential correlation between TIMP1 expression levels and either overall survival in ovarian cancer patients or the expression of ovarian cancer stemness markers. In particular, The Cancer Genome Atlas (TCGA) dataset was used to estimate ovarian cancer patients’ overall survival, based on Gene Chip mRNA expression data. To elaborate the data, the Kaplan–Meier Plotter was employed. Depending on the patients under consideration (all patients, paclitaxel-treated, platinum-treated, or treated with both) the parameter “restrict analysis to treatment groups—chemotherapy” was set. The selected cut-off value was “auto-selected best cut-off”. A *p*-value of < 0.05 was considered statistically significant. The false discovery rate was 50%.

For the GEPIA2 analysis, an OV dataset was used to check the correlation between TIMP1 and ALDH1A1, CD44, NANOG, and ABCG2 (4 signatures) expression in cancer tissues. The ‘correlation analysis’ function was applied, using the Pearson correlation coefficient for comparison. The cut-off value was set to “median cut-off”, and a *p*-value of < 0.05 was considered statistically significant.

### 2.17. Statistical Analysis

The statistical analyses were carried out using Excel 2013 and GraphPad Prism 5 for Windows. Two-tailed, unpaired Student’s *t*-tests were used to determine the significance for all experiments. GraphPad Prism 5’s “nonlinear fit of normalized and transformed data” function was used to determine the IC50 for all ovarian cancer models. For all the analyses, a probability lower than 5% was accepted as significant (*p* < 0.05). All results are expressed as the mean and standard error of the mean (SEM) of at least three different experiments.

## 3. Results

### 3.1. Ovarian Cancer Spheroids Are Characterized by Low Sensitivity to Standard Therapy and Resistance to Apoptosis

OC spheroids were generated from two HGSC cell lines (Ovcar5 and Ovcar8) [18,19,20,21,22], using an anchorage-independent culture system. In our previous work [6], we confirmed the expression of OC stemness markers ALDH, CD44, ABCG2, and NANOG as well as the spheroids’ clonogenic nature. These results allowed us to validate our spheroids as OC stem cells [6].

Since CSCs are considered the primary cause of standard therapy failure, we analyzed the sensitivity of the spheroids to paclitaxel and carboplatin treatments by determining their IC50, comparing them to bidimensional cultures of the same cell lines. Spheroids displayed low sensitivity to paclitaxel and carboplatin treatments (Figure S1A,B).

We also analyzed the apoptotic profile of the spheroids by comparing the expression of Bax and Bcl-XL to 2D cultures. Ovcar5 and Ovcar8 spheroids exhibited increased expression of the anti-apoptotic marker Bcl-XL and reduced expression of Bax (Figure S1C,D).

These data, together with stemness marker expression and genomic data [6], confirmed the stem-like properties of our spheroids.

### 3.2. Ovarian Cancer Spheroids Show Increased TIMP1 Expression Levels

**Figure 1:**
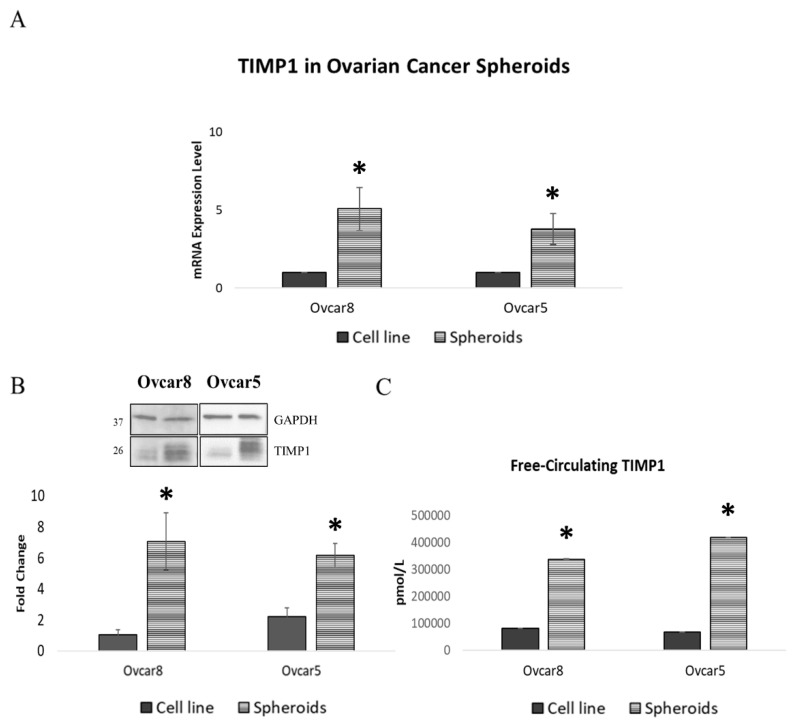
Characterization of TIMP1 expression in ovarian cancer spheroids. (**A**) Real-time PCR analysis of TIMP1 expression levels in OC spheroids. Quantification is expressed as fold change between each spheroid and its corresponding cell line. (**B**) Western blot analysis and quantification for TIMP1 expression in OC spheroids (File S1). GAPDH was used as a housekeeping protein. (**C**) Expression of TIMP1 in culture medium quantified by ELISA assay in OC spheroids and the corresponding cell line. Images are representative of at least three independent experiments; Student’s *t*-test was used to compare the groups; * *p*-value < 0.05 was considered statistically significant.

To explore how OC spheroids contribute to progression and metastasis, we evaluated the expression of matrix metalloproteinases (MMPs) and their inhibitors. Surprisingly, both spheroids exhibited increased TIMP1 (Tissue Inhibitor of Metalloproteinases-1) expression at both the transcript and protein levels (Figure 1A,B, File S1). Furthermore, an ELISA assay on culture supernatants revealed that spheroids displayed increased TIMP1 release after acquiring stemness potential (Figure 1C), confirming the secreted nature of TIMP1.

### 3.3. High TIMP1 Expression Correlates with Worse Overall Survival in Patients

**Figure 2:**
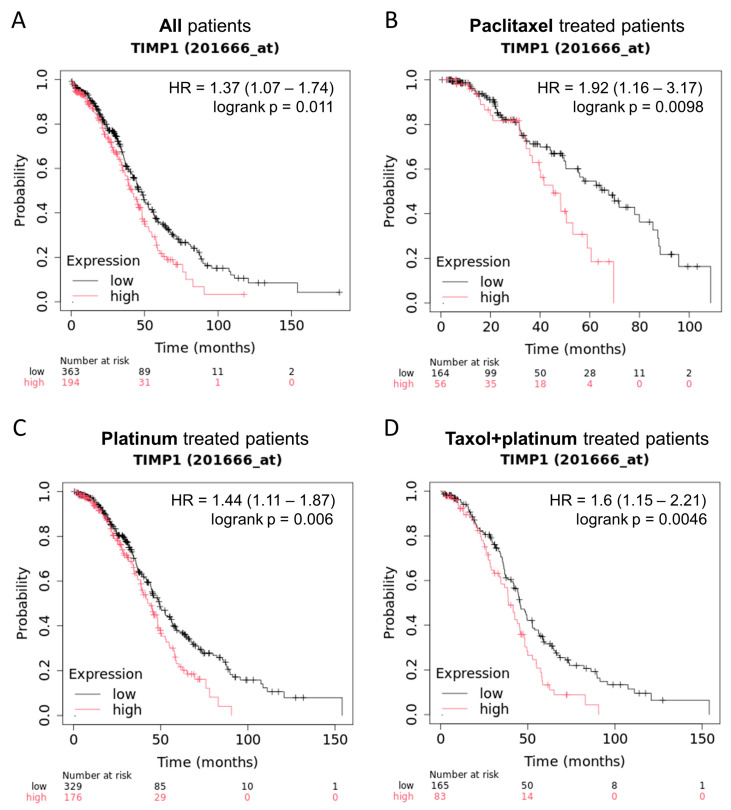
Kaplan–Meier Plotter correlation analysis between patients’ overall survival and TIMP1 expression levels in TCGA dataset. (**A**) Correlation between patients’ overall survival and TIMP1 expression. (**B**) Correlation between paclitaxel-treated patients’ overall survival and TIMP1 expression. (**C**) Correlation between platinum-treated patients’ overall survival and TIMP1 expression. (**D**) Correlation between taxol+platinum-treated patients’ overall survival and TIMP1 expression. The log-rank test was used to compare the groups; *p*-value < 0.05 was considered statistically significant.

Based on previous data and the poorly defined role of TIMP1 in OC progression, we used the Kaplan–Meier Plotter (https://kmplot.com/analysis/, last access on 19 April 2025) web server to elaborate data from The Cancer Genome Atlas database (TCGA). High TIMP1 expression correlated with worse overall survival, suggesting its potential as a prognostic marker in OC progression (Figure 2A). This correlation appeared to be independent of treatment with paclitaxel, platinum, or their combination (taxol + platinum) (Figure 2B–D).

### 3.4. Chemoresistant Ovarian Cancer Cell Lines Show Increased TIMP1 Expression

To investigate the role of TIMP1 in response to standard therapy, we developed two chemoresistant OC cell lines by exposing Ovcar8 and Ovcar5 cells to increasing doses of paclitaxel and carboplatin. First, we characterized these models by evaluating their sensitivity to standard therapy, comparing them to the corresponding parental cell lines. Dose/response experiments demonstrated acquired resistance to standard therapy in both Ovcar8- and Ovcar5-resistant cell lines (Figure S2A,B). To further validate our models, we analyzed the expression levels of the stemness markers ALDH1, CD44, ABCG2, and NANOG using real-time PCR. As expected, our results confirmed the acquisition of stemness characteristics in the resistant cells (Figure S2C).

**Figure 3:**
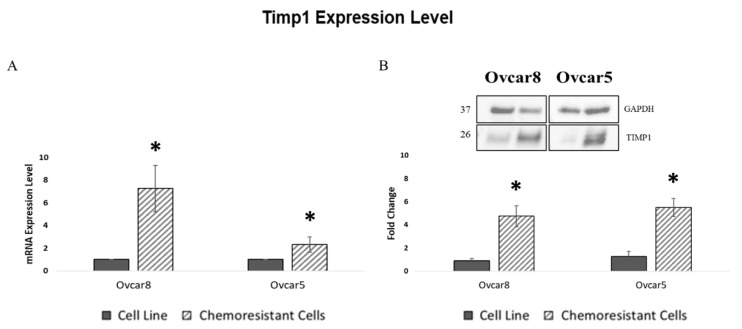
TIMP1 expression in chemoresistant models. (**A**) Real-time PCR analysis of TIMP1 expression level in OC chemoresistant cells. Quantification is expressed as fold change between each chemoresistant model and its corresponding cell line. (**B**) Western blot analysis and quantification for TIMP1 expression in OC-resistant cells. GAPDH was used as a housekeeping protein. The images are representative of at least three independent experiments (File S1); Student’s *t*-test was used to compare the groups; * *p*-value < 0.05 was considered statistically significant.

To assess if the acquisition of stemness properties and therapy resistance correlates with TIMP1 expression in resistant cell line, as observed in our spheroid models, we performed gene expression as well as Western blot analyses. Our results demonstrate that both the Ovcar8- and Ovcar5-resistant cell lines exhibited increased TIMP1 expression compared to their parental counterparts (Figure 3, File S1).

### 3.5. Anoikis-Resistant Cell Lines Display Increased TIMP1 Expression

**Figure 4:**
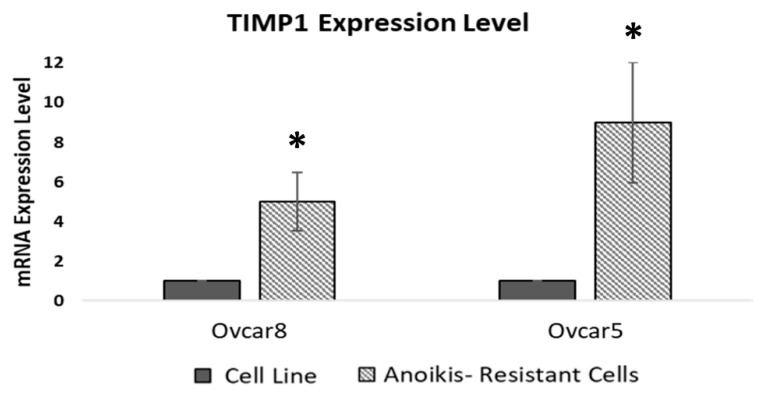
TIMP1 expression in anoikis-resistant models. Real-time PCR analysis of TIMP1 expression levels in OC anoikis-resistant cells. Quantification is expressed as fold change between each anoikis-resistant model and its corresponding cell line. Images are representative of at least three different experiments; Student’s *t*-test was used to compare the groups; * *p*-value < 0.05 was considered statistically significant.

OC spheroids were characterized by their ability to grow in an anchorage-independent system, a phenomenon known as anoikis resistance. To explore whether anoikis resistance is related to TIMP1 expression, we developed anoikis-resistant OC models by exposing Ovcar8 and Ovcar5 cells to suspension stress cycles, alternated with attached recovery growth. Real-time PCR analysis confirmed the higher expression of stemness markers (ALDH1, CD44, ABCG2, and NANOG) in the anoikis-resistant cells (Figure S3). We then analyzed TIMP1 expression in the anoikis-resistant models and found that both Ovcar8- and Ovcar5-resistant cells exhibited increased TIMP1 levels compared to the parental cell lines, suggesting a pivotal role of TIMP1 in anoikis resistance (Figure 4).

### 3.6. TIMP1 Overexpression Is Linked to Therapy and Anoikis Resistance

**Figure 5:**
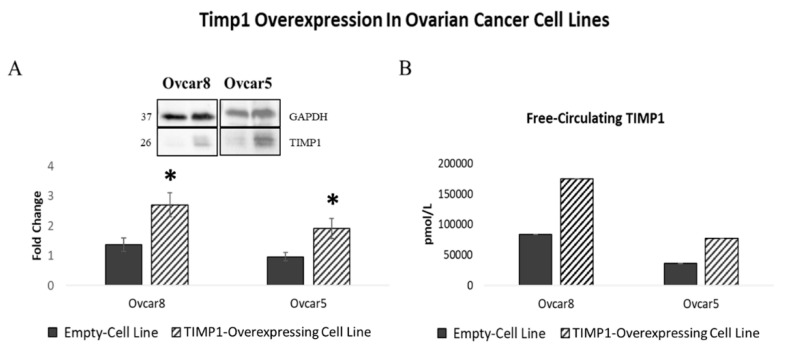
Characterization of TIMP1 overexpression. (**A**) Western blot analysis and quantification of TIMP1 expression in TIMP1-overexpressing cells (File S1). Anti-GAPDH was used as a housekeeping protein. Western blot bands: left, empty vector; right, TIMP1 vector. (**B**) TIMP1 expression in the culture medium quantified by ELISA assay in TIMP1-overexpressing cells and the corresponding controls. Images are representative of at least three independent experiments. Student’s *t*-test was used to compare the groups; * *p*-value < 0.05 was considered statistically significant.

To further investigate the role of TIMP1 in OC stem cells and its involvement in OC progression, we stably overexpressed TIMP1 in the Ovcar8 and Ovcar5 cell lines to determine whether increased TIMP1 expression could replicate the phenotypes observed in previous models. We first assessed the efficacy of transfection at both transcript and protein levels. Real-time PCR and WB analyses confirmed successful TIMP1 overexpression in the OC cell lines (Figures S4 and 5A, File S1). As observed in the spheroid model, we investigated whether elevated TIMP1 expression was associated with increased release into the microenvironment. ELISA assays on TIMP1-overexpressing Ovcar8 and Ovcar5 cells, compared to control cells (Ovcar8 and Ovcar5 transfected with an empty vector), indicated a trend toward higher TIMP1 secretion in the TIMP1-transfected lines, although this difference was not statistically significant (Figure 5B).

**Figure 6:**
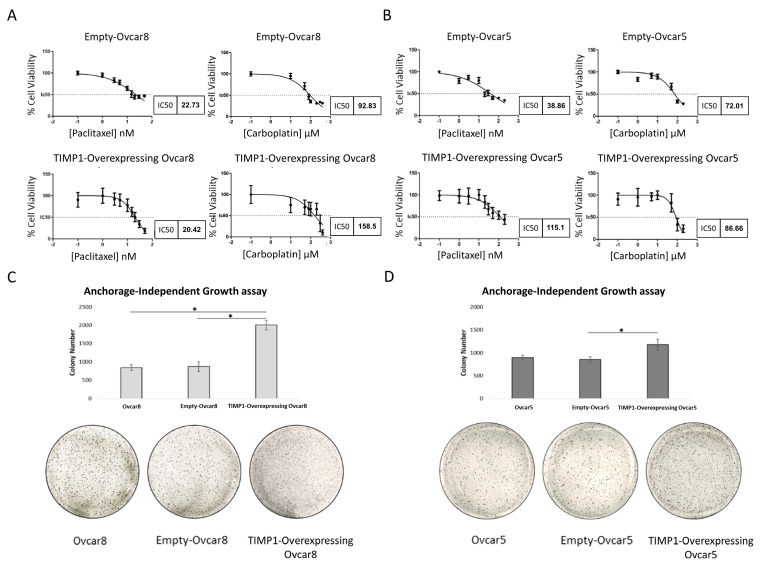
Characterization of TIMP1-overexpressing cells in response to therapy and anoikis resistance. (**A**,**B**) IC50 values of TIMP1-overexpressing cells and their corresponding control cells were calculated using GraphPad Prism. An MTT assay was performed 72 h after treatment with increasing doses of paclitaxel and carboplatin. (**A**) IC50 values of TIMP1-overexpressing Ovcar5 cells for paclitaxel and carboplatin treatment. (**B**) IC50 values of TIMP1-overexpressing Ovcar8 cells for paclitaxel and carboplatin treatment. (**C**,**D**) Soft agar colony formation assay performed on TIMP1-overexpressing cells. Empty and untreated cell lines were used as controls. After 2 weeks, colonies were stained with MTT and quantified based on colony size and number using IMAGEJ software. Magnification 4×. (**C**) Anchorage-independent growth in TIMP1-overexpressing Ovcar8 cells. (**D**) Anchorage-independent growth in TIMP1-overexpressing Ovcar5 cells. Images are representative of at least three independent experiments. Student’s *t*-test was used to compare the groups; * *p*-value < 0.05 was considered statistically significant.

To further explore the role of TIMP1 in response to standard therapy, we determined the IC50 values of TIMP1-transfected Ovcar8 and Ovcar5 cells for paclitaxel and carboplatin treatments, as compared to control cells. TIMP1-overexpressing Ovcar5 cells exhibited a significant reduction in sensitivity to standard therapy, compared to the controls, while Ovcar8 showed reduced sensitivity only to carboplatin after TIMP1 overexpression (Figure 6A,B). Then, to confirm TIMP1’s involvement in anoikis resistance, we performed a soft agar colony formation assay on TIMP1-transfected ovarian cells and their respective counterparts. All TIMP1-overexpressing cells exhibited increased anchorage-independent growth compared to control cells, confirming the acquisition of resistance to anoikis following TIMP1 overexpression (Figure 6C,D).

### 3.7. TIMP1 Overexpression Increases Ovarian Cancer Cell Migration

**Figure 7:**
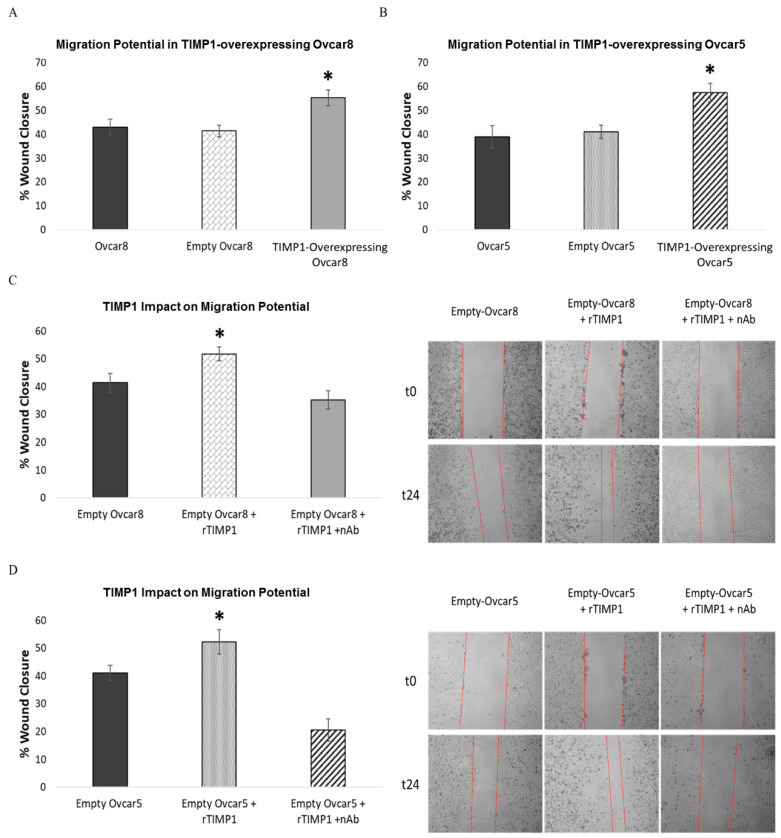
Characterization of TIMP1-overexpressing cells in response to migration. (**A**,**B**) Wound healing assay on TIMP1-overexpressing OC cells. Parental OC cells and empty cells were used as control. For wound closure quantification, the software IMAGEJ was used. The results are expressed in terms of the percentage of wound closure. Images were acquired 24 h after the wound healing assay was performed and represent at least three experiments. (**A**) Wound healing assay on Ovcar8 cells. (**B**) Wound healing assay on Ovcar5 cells. (**C**,**D**) Wound healing assay on empty ovarian cancer cells treated with TIMP1-recombinant protein alone or in the presence of TIMP1-neutralizing antibody. Magnification 4×. (**C**) Wound healing assay on empty Ovcar8 cells. (**D**) Wound healing assay on empty Ovcar5 cells. For wound closure quantification, the software ImageJ was used. The results are expressed in terms of the percentage of wound closure. Images were acquired 24 h after the wound assay was performed and represent at least three experiments; Student’s *t*-test was used to compare the groups; * *p*-value < 0.05 was considered statistically significant.

Dysregulation of anoikis, particularly resistance to it, plays a key role in tumor metastasis [23]. On this basis, to evaluate whether TIMP1 is involved in the migration capability of OC cells, we performed a wound healing assay on TIMP1-overexpressing Ovcar8 and Ovcar5 cells, comparing them to empty cells and parental cell lines. TIMP1-overexpressing cells showed increased migration potential compared to controls (Figure 7A,B).

To confirm this, we replicated the assay on empty-vector-transfected Ovcar8 and Ovcar5 treated with human soluble recombinant TIMP1 protein with or without a TIMP1-neutralizing antibody. We chose this strategy as TIMP1 is a secreted protein, and its mechanism involves alternating cycles of release and reuptake. The results confirmed that TIMP1 overexpression enhanced OC cell migration. In fact, empty cells showed increased migration only in the presence of recombinant TIMP1 protein (Figure 7C,D).

### 3.8. TIMP1 Overexpression in Ovarian Cancer Cell Lines Is Related to Stem Cell Marker Expression

**Figure 8:**
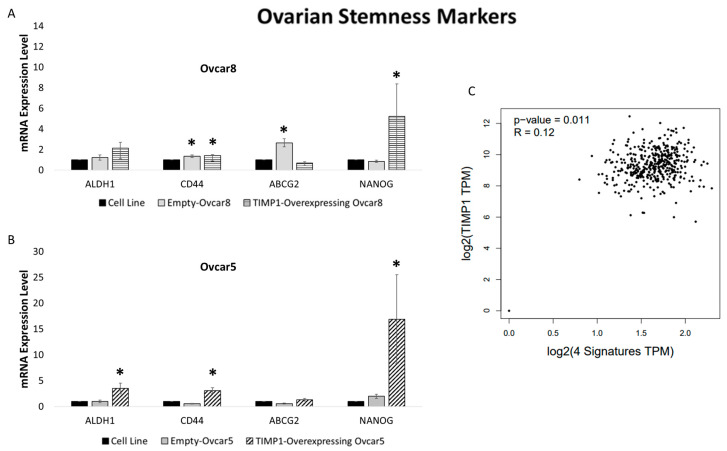
TIMP1 expression and OC stemness markers. (**A**,**B**) Real-time PCR analysis of ovarian cancer stemness marker expression levels in TIMP1-overexpressing cells. Empty and untreated cell lines were used as control. Quantification is expressed as fold change. (**A**) Ovcar8 cells. (**B**) Ovcar5 cells. Images are representative of at least three independent experiments. Student’s *t*-test was used to compare the groups; * *p*-value < 0.05 was considered statistically significant. (**C**) GEPIA2 web server correlation analysis of ovarian stemness marker expression and TIMP1 expression levels. Pearson correlation coefficient was used for comparison; * *p*-value < 0.05 was considered statistically significant.

To assess whether TIMP1 correlated with the acquisition of stemness potential, we analyzed the expression of previously analyzed [6] ovarian stemness markers, ALDH1, CD44, ABCG2, and NANOG, in TIMP1-overexpressing cells and controls. TIMP1 overexpression significantly increased ALDH1, CD44, and NANOG expression in Ovcar5 cells, while in Ovcar8 cells, it significantly enhanced NANOG and CD44 expression and showed a trend toward increased ALDH1 expression, although not reaching statistical significance (Figure 8A,B). Further analysis of the TCGA-OV dataset expression data via the GEPIA2 web server revealed a significant correlation between TIMP1 and stemness markers also in OC samples (Figure 8C).

### 3.9. TIMP1-Overexpressing Ovcar5 Cells Exhibit Powered Metastatic Potential In Vivo

**Figure 9:**
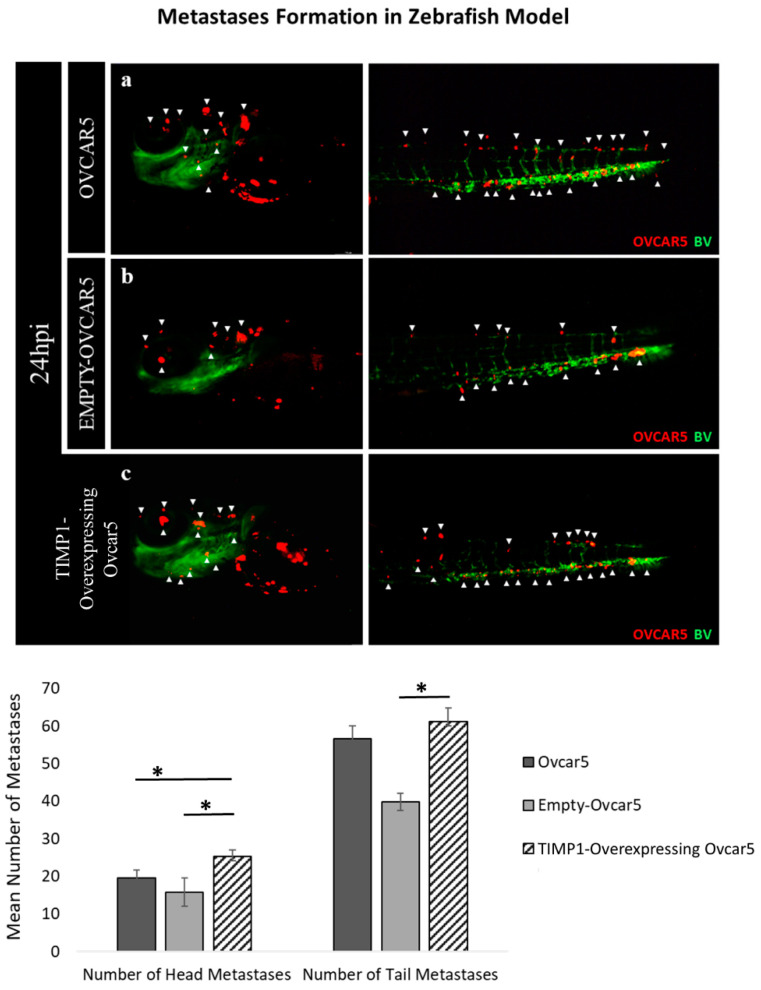
Zebrafish models. (**a**) Ovcar5 wild type, (**b**) empty Ovcar5, and (**c**) TIMP1-overexpressing Ovcar5 cells were injected into the perivitelline space (PVS) of 48 h post-fertilization Tg(fli1:EGFP) zebrafish larvae, which display green fluorescent blood vessels (BVs). The cells were fluorescently labeled with CM-Dil cell tracker (indicated by white arrows), and zebrafish tumors xenografts were analyzed at 24 h post-injection. Metastatic cancer cells are indicated in the zebrafish head and tail with white arrowheads. Images are representative of at least three different experiments. Student’s *t*-test was used to compare the groups; * *p*-value < 0.05 was considered statistically significant.

To validate our in vitro results and determine whether TIMP1 overexpression confers an advantage in migration, dissemination, and metastatic potential, we generated zebrafish xenotransplantation models. Ovcar5 cells, transfected with either a TIMP1 vector or empty vector and labeled with the red cell tracker CM-Dil, were injected into the perivitelline space of zebrafish larvae. After 24 h, larvae were analyzed using a fluorescence stereomicroscope. TIMP1-overexpressing Ovcar5 showed significantly enhanced metastatic potential compared to the empty Ovcar5 cells, regardless of the metastatic site and an increased tropism for head metastasis formation compared to the Ovcar5 cells (Figure 9).

### 3.10. TIMP1 Overexpression Significantly Alters the Transcriptome of Ovarian Cancer Cells

**Figure 10:**
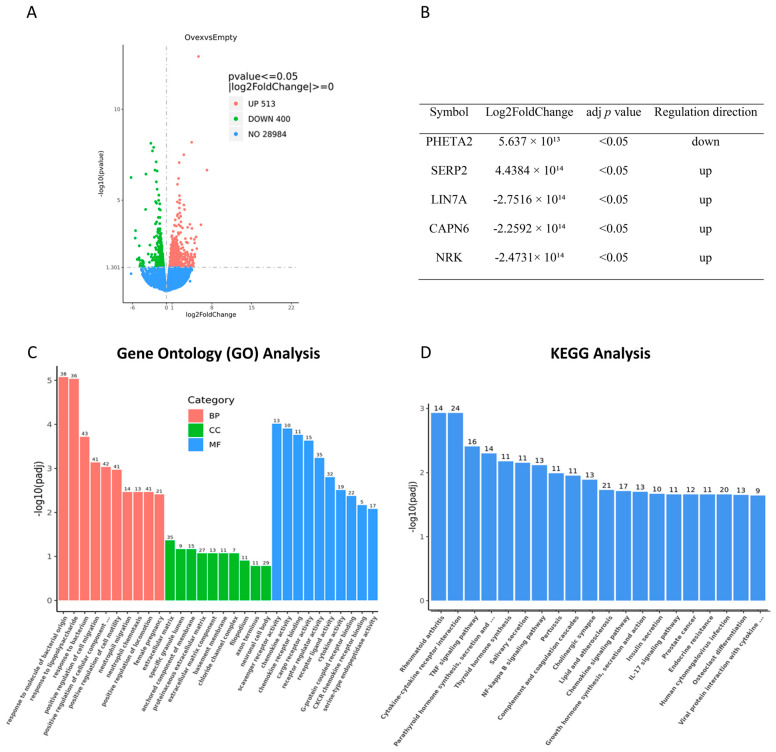
Bulk RNA sequencing of TIMP1-overexpressing Ovcar5 cells and empty-vector-transfected Ovcar5 cells (control). (**A**) Volcano plots showing the overall distribution of differentially expressed genes. (**B**) Table listing the top dysregulated genes. (**C**) Gene Ontology (GO) enrichment analysis displaying the 30 most significantly enriched terms in TIMP1-overexpressing Ovcar5 cells; BP, CC, and MF represent biological processes, cellular components, and molecular functions, respectively. The x-axis indicates the GO term, and the y-axis represents the significance level of enrichment, expressed as −log10(padj). (**D**) KEGG pathway enrichment analysis showing the 20 most significantly enriched KEGG pathways in TIMP1-overexpressing Ovcar5 cells; the x-axis indicates the ratio of the number of differentially expressed genes associated with the KEGG pathway to the total number of differentially expressed genes, and the y-axis indicates the KEGG pathway. An adjusted *p*-value (padj) <0.05 was considered statistically significant.

To investigate the gene expression profile following TIMP1 overexpression, RNA-Seq analysis was performed on TIMP1-overexpressing and empty-vector-transfected Ovcar5 cells as controls. A total of 913 differentially expressed genes (DEGs) were identified, with 513 upregulated and 400 downregulated (Figure 10A). Notably, *SERP2*, *LIN7A*, *CAPN6*, and *NRK* were significantly upregulated, while *PHETA2* was downregulated (Figure 10B). Gene Ontology (GO) analysis revealed enrichment in processes related to cell migration, motility, and neutrophil migration, as well as extracellular matrix and membrane-anchored components (Figure 10C). Collectively, these findings emphasize the contribution of TIMP1 overexpression to the regulation of cancer cell migration.

KEGG analysis revealed significant enrichment in pathways such as rheumatoid arthritis, cytokine–cytokine receptor interactions, tumor necrosis factor signaling, and NF-kappa B signaling (Figure 10D). These preliminary data suggest that TIMP1 overexpression significantly alters the transcriptomic landscape of OC cells, influencing critical pathways involved in cell migration and inflammation. TIMP1’s role in these processes highlights its potential as a key regulator in OC progression and as a promising therapeutic target for future interventions.

## 4. Discussion

According to the CSC theory, only a fraction of cells drives tumor progression, metastasis, and relapse. Standard therapies temporarily improve patient conditions, however, without affecting this specific population [24]. OC is an ideal example of a CSC-driven neoplasm, characterized by high aggressiveness and cells that spread within the abdominal cavity [25]. OC spheroids in malignant ascites represent the subpopulation responsible for ovarian cancer dissemination, treatment resistance, and poor prognosis [26]. Hence, studying spheroid models in vitro provides a clinically relevant strategy, recapitulating stemness properties, including the development of chemoresistance and anoikis resistance, but also represents a promising tool for identifying novel biomarkers for predicting OC behavior [11,27].

In this manuscript, we described, for the first time, the overexpression of TIMP1 in OC stem cells, highlighting its potential usefulness as a prognostic marker for OC. Spheroids were previously isolated and characterized based on the expression of stemness markers, clonogenicity, and genomic profile [6,28]. Given the intrinsic resistance of CSCs to apoptosis, we investigated the expression of apoptotic markers, demonstrating the upregulation of Bcl-XL and downregulation of Bax (Figure S1C,D), key modulators of apoptosis [29]. Notably, our spheroid model faithfully replicated the acquired resistance to carboplatin and paclitaxel, reflecting the role of OC stem cells in primary chemotherapy failure and tumor recurrence (Figure S1A,B) [30].

OC spheroids showed TIMP1 overexpression (Figure 1A,B). TIMP1 is one of the main elements involved in maintaining tissue integrity but has recently emerged as a key actor implicated in various cancers [31]. This contradicts the known role of TIMP1 as a natural inhibitor that can modulate the invasive and metastatic capacity of tumor cells [13]. Despite these considerations, TIMP1 overexpression has been linked to tumor proliferation, metastasis, and anti-apoptotic signaling in colon cancer [32] and poor prognosis in breast cancer [33]. Increased TIMP1 expression has also been reported for OC, suggesting a chemo-protective role in chemotherapy-resistant cells [34]. TCGA data corroborated a significant correlation between TIMP1 expression and poor overall survival, regardless of the treatment administered (Figure 2). Abreu et al. also reported increased TIMP1 expression in ovarian ascites, indicating its presence in the tumor’s secretome rather than its retention in tissues [31]. Additionally, ELISA assays conducted on our spheroid models revealed elevated TIMP1 levels in the surrounding microenvironment (Figure 1C), reinforcing its potential as a biomarker for disseminated disease.

To investigate TIMP1’s role in therapy response, we developed two chemoresistant OC cell lines (Figure S2A,B). Both lines acquired chemoresistance, associated with increased stemness and elevated TIMP1 expression (Figures S2C and 3). However, while the link between resistance to therapy and stemness was already well established [35], the increased TIMP1 expression strongly suggests its direct involvement in regulating these properties. These data were further confirmed by TIMP1 overexpression (Figures S4 and 5A), which conferred resistance to platinum-based chemotherapy and paclitaxel (Figure 6A,B), consistent with findings by Sonego et al. [36]. TIMP1 has also been linked to paclitaxel resistance, where higher TIMP1 levels in tumor tissues were associated with poor response to paclitaxel-based therapy in breast cancer [37], though this has not been observed in other cancers [38,39].

The relationship between TIMP1 expression and acquired stemness properties was also supported by the development of anoikis-resistant cells. These cells showed increased expression of ovarian stemness markers following the acquisition of anchorage-independent growth (Figure S3) and exhibited increased TIMP1 expression (Figure 4). Anoikis, a form of programmed cell death triggered by detachment from the extracellular [40], is crucial for supporting cancer cell survival across the systemic circulation and their dissemination to secondary sites [41]. It is also a key factor in spheroid formation in ovarian ascites, where OC cells need to aggregate to avoid anoikis [42]. TIMP1’s involvement in anoikis resistance has been documented in melanoma cells, where it enabled survival and growth under suspension conditions [42]. Our findings align with this, as TIMP1-transfected cell lines exhibited enhanced anoikis resistance and greater growth in soft agar (Figure 6C,D).

Metastasis represents the greatest challenge in the clinical management of cancer and consists in a multistep process, also relying on migration and invasion ability [43]. TIMP1 promoted migration in OC cells, with TIMP1-overexpressing cells demonstrating enhanced migration (Figure 7A,B). Recombinant TIMP1 treatment further enhanced migration in empty transfected cells, an effect that was inhibited by a TIMP1-neutralizing antibody (Figure 7C,D). These results align with studies linking TIMP1 overexpression to enhanced migration in leukemia [44].

Zebrafish models have emerged as valuable tools for studying cell invasion and extravasation within an intact circulatory system in a shorter period compared to murine models [45]. Furthermore, these models enable the evaluation of tumor biology, particularly in a preclinical testing, validating drug combinations and screening for drug sensitivity [46]. This is particularly relevant in ovarian cancer research, where generating patient-derived xenograft (PDX) models in mice is challenging due to a low success rate of only 10–20% [47]. Consistent with these advantages, our in vivo zebrafish models confirmed TIMP1’s role in promoting cancer cell dissemination, with zebrafish injected with TIMP1-overexpressing cells showing enhanced invasion potential compared to controls (Figure 9). These findings are consistent with Abreu et al., who showed that TIMP1-deficient breast cancer cells had lower proliferation rates in vivo, both in zebrafish embryos and orthotopic xenografts in immunosuppressed mice [48]. Similarly, Cheng et al. demonstrated that blocking TIMP1 in a mouse model of triple-negative breast cancer impaired tumor growth [49].

RNA sequencing of Ovcar5 cells revealed significant changes in gene expression following TIMP1 overexpression (Figure 10A), with GO analysis highlighting an impact on cell motility (Figure 10C). These findings corroborate our in vitro results and align with Chang et al., demonstrating a positive effect of TIMP1 on cell migration, invasion, and colonization in lung cancer [50]. Differential expression analysis revealed significant increases in SERP2, LIN7A, CAPN6, and NRK following TIMP1 overexpression (Figure 10B). Among these, LIN7A (lin-7 homolog A) plays a role in proliferation, migration, and invasion specifically in OC, where it functions as an oncogene downstream of the CASC9/miR-758-3p axis [51]. Similarly, CAPN6 (calpain 6), a non-proteolytic calpain involved in cell stability and apoptosis inhibition, has been implicated in ovarian tumorigenesis, potentially through the activation of inflammatory and survival pathways [52,53]. While direct studies in OC are more limited to SERP2 and NRK, their upregulation in this context suggests a possible role in inflammation-related signaling and tumor-stroma interaction, consistent with the inflammatory enrichment observed in our KEGG analysis.

In summary, our data suggest that TIMP1 overexpression significantly alters the gene expression profile of OC cells. The RNA sequencing results support previous findings, emphasizing TIMP1’s role in enhancing cell migration and invasion. However, further validation with additional ovarian cancer cell lines is necessary to confirm these observations.

## 5. Conclusions

In conclusion, this study highlights the role of CSC-secreted TIMP1 in ovarian cancer progression, therapy resistance, and metastasis. TIMP1 transfection in OC cells not only restored the spheroid phenotype, enhancing resistance to standard therapy, metastatic potential, and dissemination capabilities, but also increased ovarian stemness marker expression. Additionally, the secreted nature of TIMP1 presents it as a promising serum-free prognostic marker for OC [34], similar to its role in non-small-cell lung cancer, where plasma TIMP1 levels were able to discriminate between cancer patients and healthy controls [54]. However, additional research is needed to further clarify TIMP1’s role in OC, investigate its molecular mechanisms, interactions with other biomarkers, and evaluate its potential as a therapeutic target.

## Data Availability

The data presented in this study are available on request from the corresponding author.

## Supplementary Materials

The following supporting information can be downloaded at https://www.mdpi.com/article/10.3390/cancers17101605/s1, Figure S1: Ovarian cancer spheroid characterization; Figure S2: Chemoresistant cell characterization; Figure S3: Stemness marker expression in anoikis-resistant cells; Figure S4: TIMP1 expression in TIMP1-overexpressing cells; File S1: The original Western blot figures.
