# Supplementary material for: TIMP1 Overexpression in Ovarian Cancer Spheroids: Implications for Prognosis, Resistance, and Metastatic Potential
Source: Cancers (Basel). 2025 May 9;17(10):1605. doi: 10.3390/cancers17101605 (PMC12109905; doi:10.3390/cancers17101605)

FILE S1. The original Western blot figures.

Figure 1B

Ovcar5 cells and spheroids. The first panel shows the representative image; the others are replicates. Red: TIMP1 protein (26 kDa); blue: GAPDH protein (37 kDa).

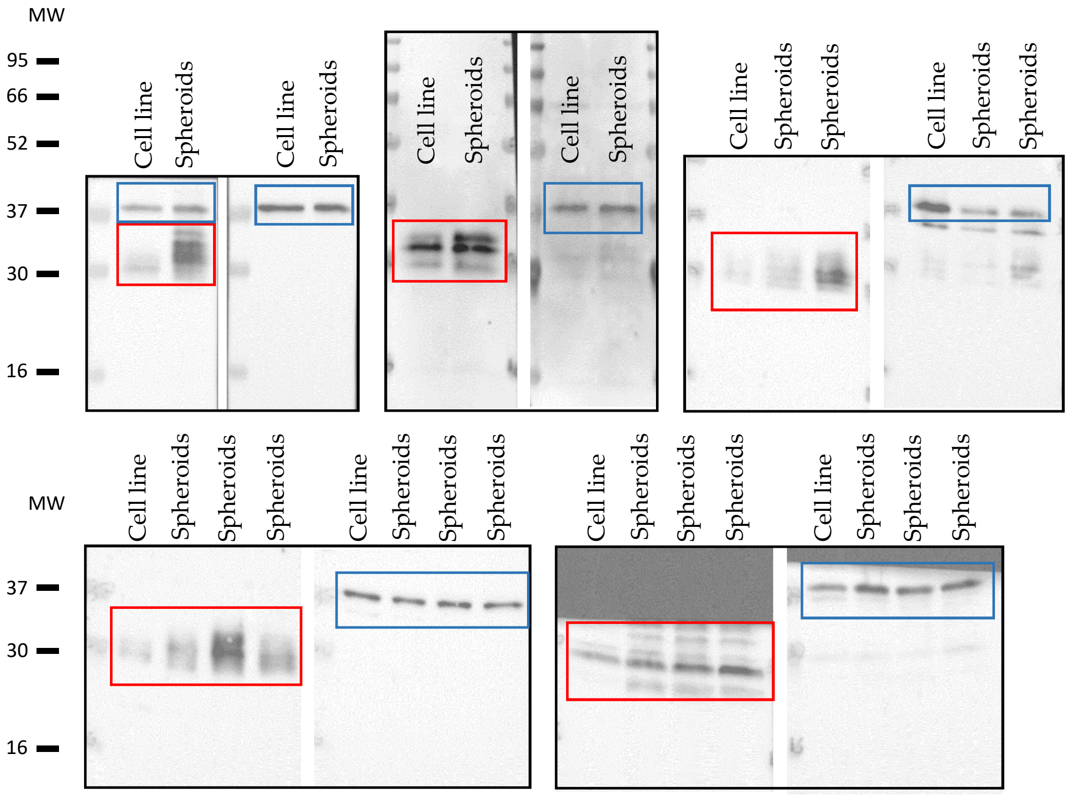

Ovcar8 cells (always on the left) and spheroids (always on the right). The first panel shows the representative image; the others are replicates. Red: TIMP1 protein (26 kDa); blue: GAPDH protein (37 kDa).

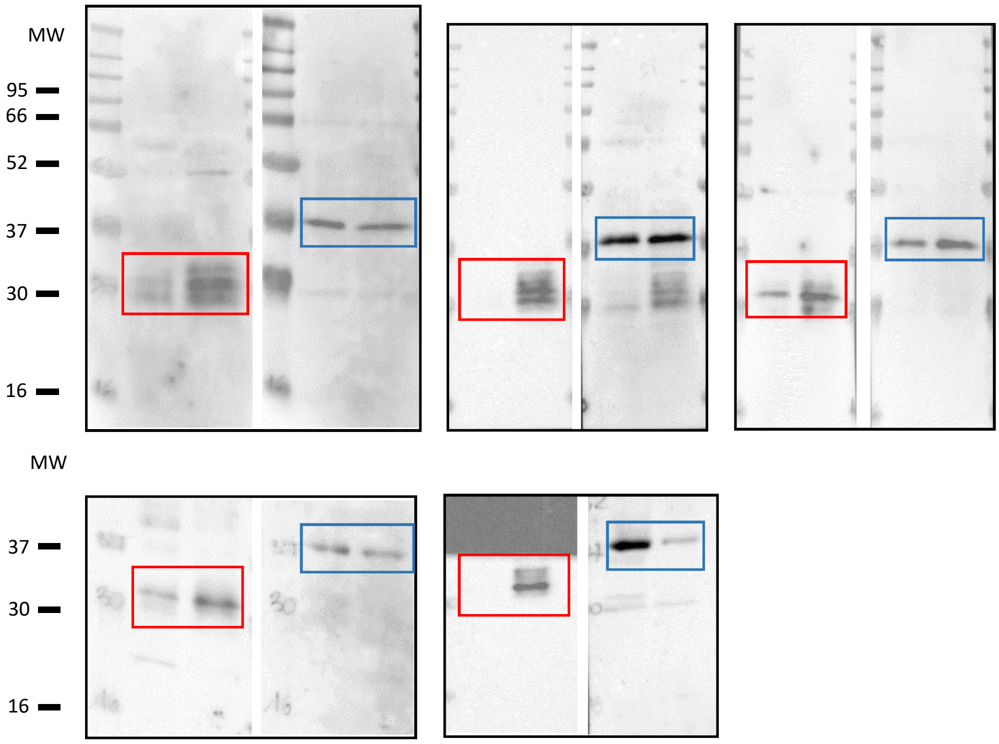

Figure 3B

Ovcar5 cells (always on the left) and chemoresistant cells (always on the right). The first panel shows the representative image; the others are replicates. Red: TIMP1 protein (26 kDa); blue: GAPDH protein (37 kDa).

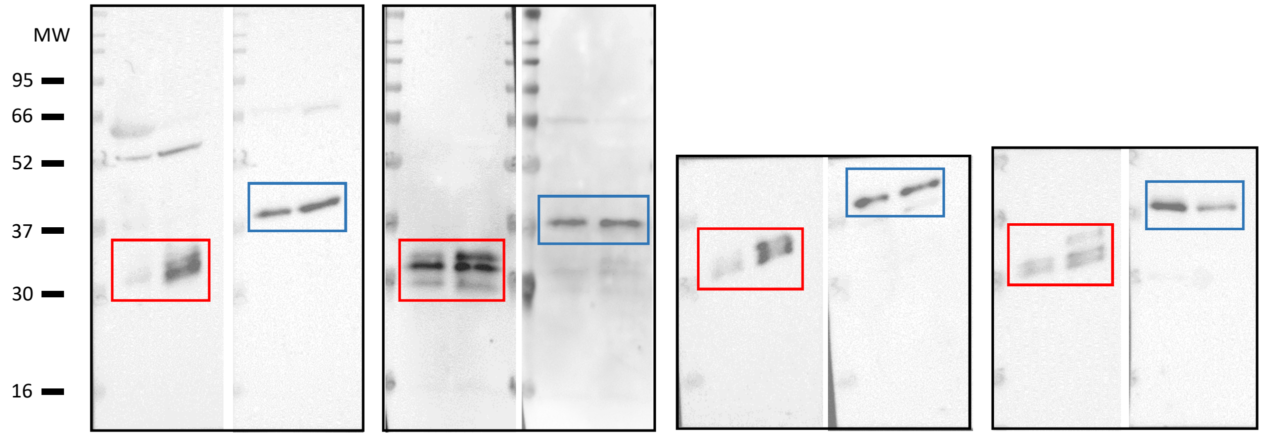

Ovcar8 cells (at different passages) and chemoresistant cells (at different passages). The first panel shows the representative image; the others are replicates. Red: TIMP1 protein (26 kDa); blue: GAPDH protein (37 kDa).

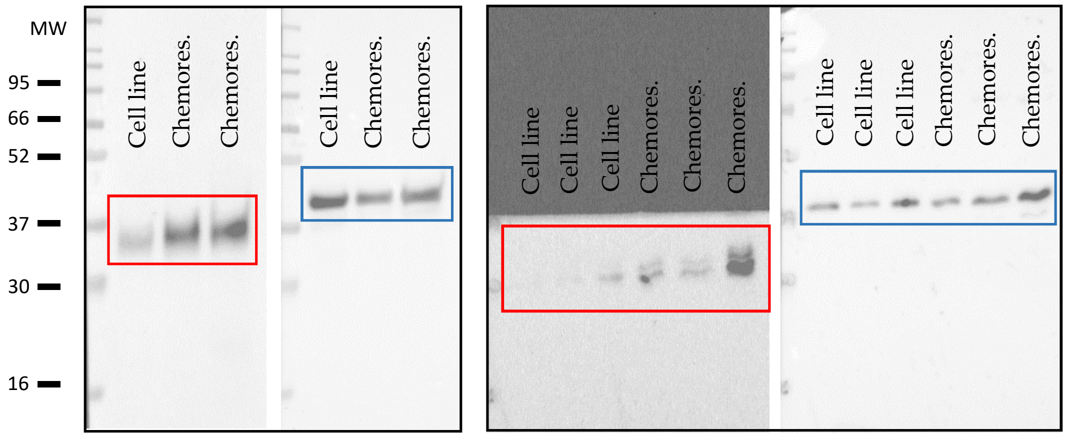

Figure 5A

Ovcar5 empty-cells and TIMP1-overexpressing cells. The first panel shows the representative image; the others are replicates. Red: TIMP1 protein (26 kDa); blue: GAPDH protein (37 kDa).

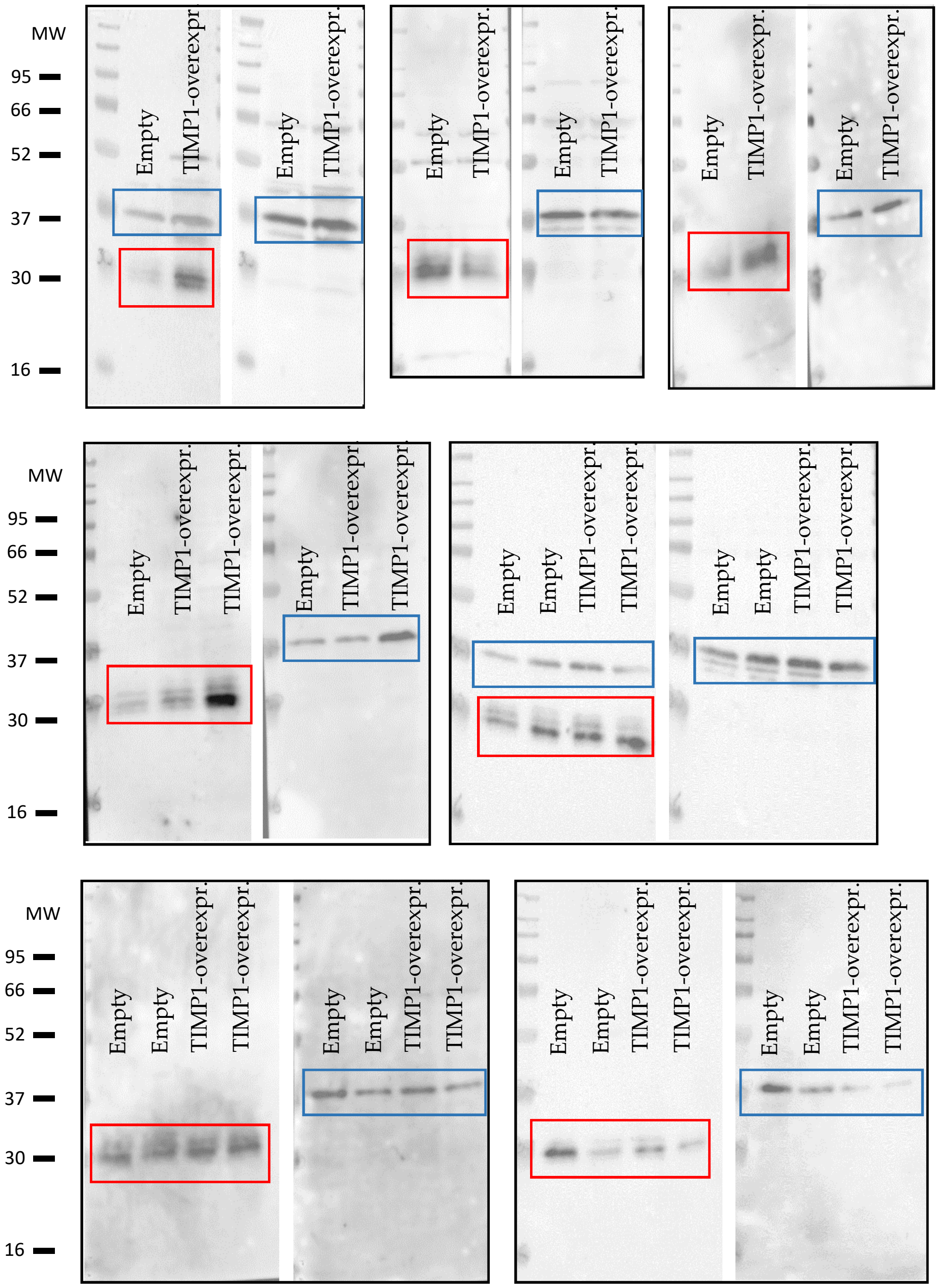

Figure 5A

Ovcar8 empty-cells and TIMP1-overexpressing cells. The first panel shows the representative image; the others are replicates. Red: TIMP1 protein (26 kDa); blue: GAPDH protein (37 kDa).

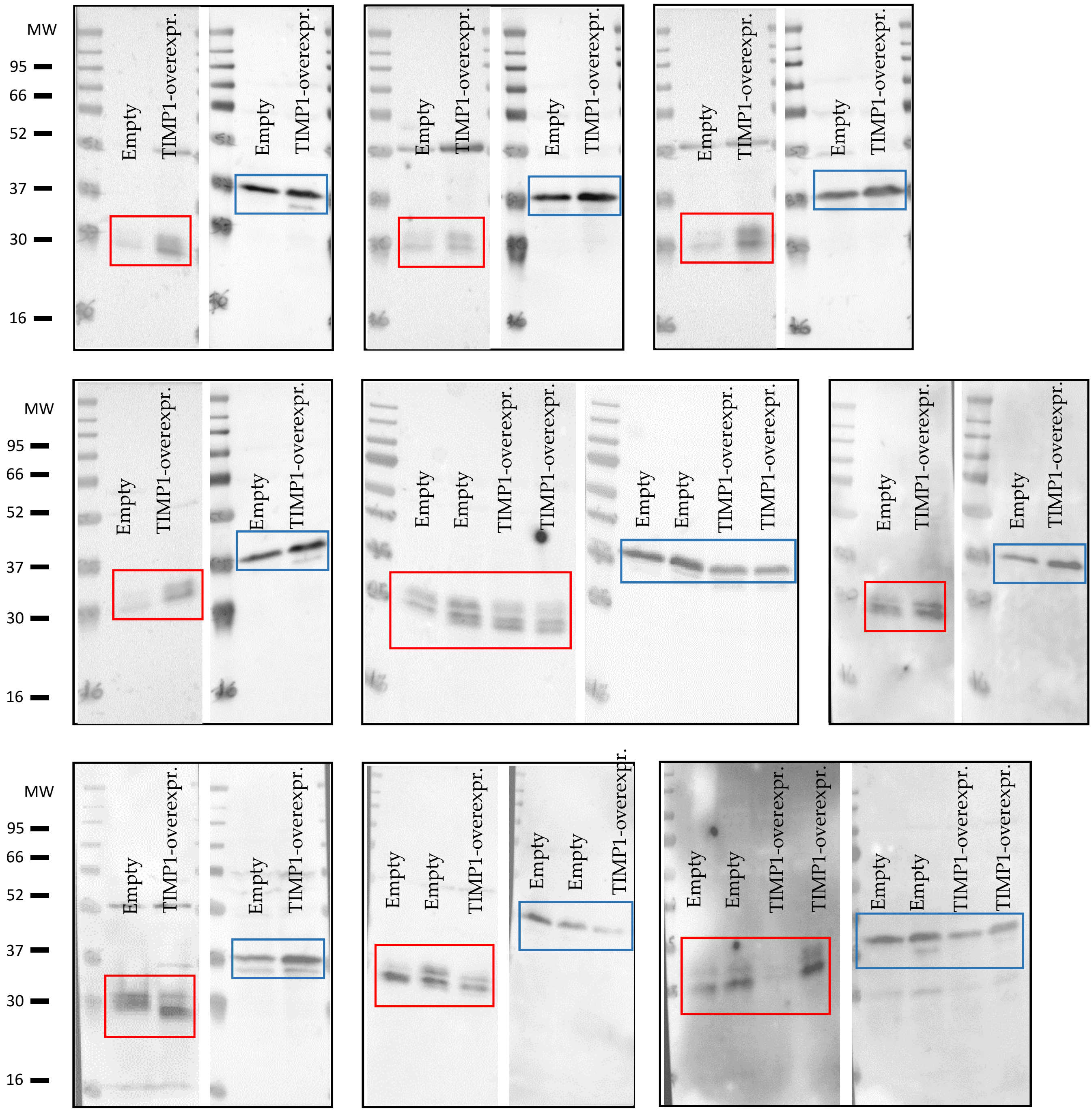

Supplement: Supplementary file 1 [file cancers-17-01605-s001.zip › File S1.pdf]
