# Supplementary material for: TIMP1 Overexpression in Ovarian Cancer Spheroids: Implications for Prognosis, Resistance, and Metastatic Potential
Source: Cancers (Basel). 2025 May 9;17(10):1605. doi: 10.3390/cancers17101605 (PMC12109905; doi:10.3390/cancers17101605)
Supplement: Supplementary file 1 [file cancers-17-01605-s001.zip › Supporting Information.pdf]

Supporting Information

Figure S1: Ovarian cancer spheroids characterization. (A-B) IC50 of Spheroids and the corresponding cell line calculated using GraphPad Prism. MTT assay was performed 72h after treatment with increasing doses of carboplatin (A) and paclitaxel (B). (C-D) Western Blot analysis and quantification for apoptotic markers expression in ovarian cancer spheroids. (C) Bcl-XL expression in Ovcar8 andOvcar5 spheroids. (D) Bax expression in Ovcar8 and Ovcar5 spheroids. Each result is reported as fold change, was compared to the corresponding cell line. GAPDH was used as housekeeping protein. Images are representative of at least three different experiments; Student's t test was used to compare the groups; * p value <0.05 was considered statistically significant.

Figure S2: Chemoresistant cells characterization. (A-B) IC50s of chemoresistant cells and the corresponding cell lines calculated using GraphPad Prism. MTT assay was performed on Chemoresistant Ovcar8 (A) and Chemoresistant Ovcar5 (B). The IC50s of the control cell line were reported in the tables. (C) Real-time PCR analysis of ovarian cancer stemness markers expression level between chemoresistant cells and their corresponding cell line. Quantification is expressed as fold change. Images are representative of at least three different experiments; Student's t test was used to compare the groups; * p value <0.05 was considered statistically significant.

Figure S3: Stemness markers expression in anoikis-resistant cells. Real-time PCR analysis of ovarian cancer stemness markers expression level between anoikis-resistant cells and their corresponding cell line. Quantification is expressed as fold change. Images are representative of at least three different experiments; Student's t test was used to compare the groups; * p value <0.05 was considered statistically significant.

Figure S4: TIMP1 expression in TIMP1-overexpressing cells. Quantification is expressed as fold change between each TIMP1-overexpressing cell line and its corresponding control (the corresponding cell line transfected with the empty vector). Images are representative of at least three different experiments; Student's t test was used to compare the groups; * p value <0.05 was considered statistically significant.
