# Supplementary figures and images for: TIMP1 Overexpression in Ovarian Cancer Spheroids: Implications for Prognosis, Resistance, and Metastatic Potential
Source: Cancers (Basel). 2025 May 9;17(10):1605. doi: 10.3390/cancers17101605 (PMC12109905; doi:10.3390/cancers17101605)

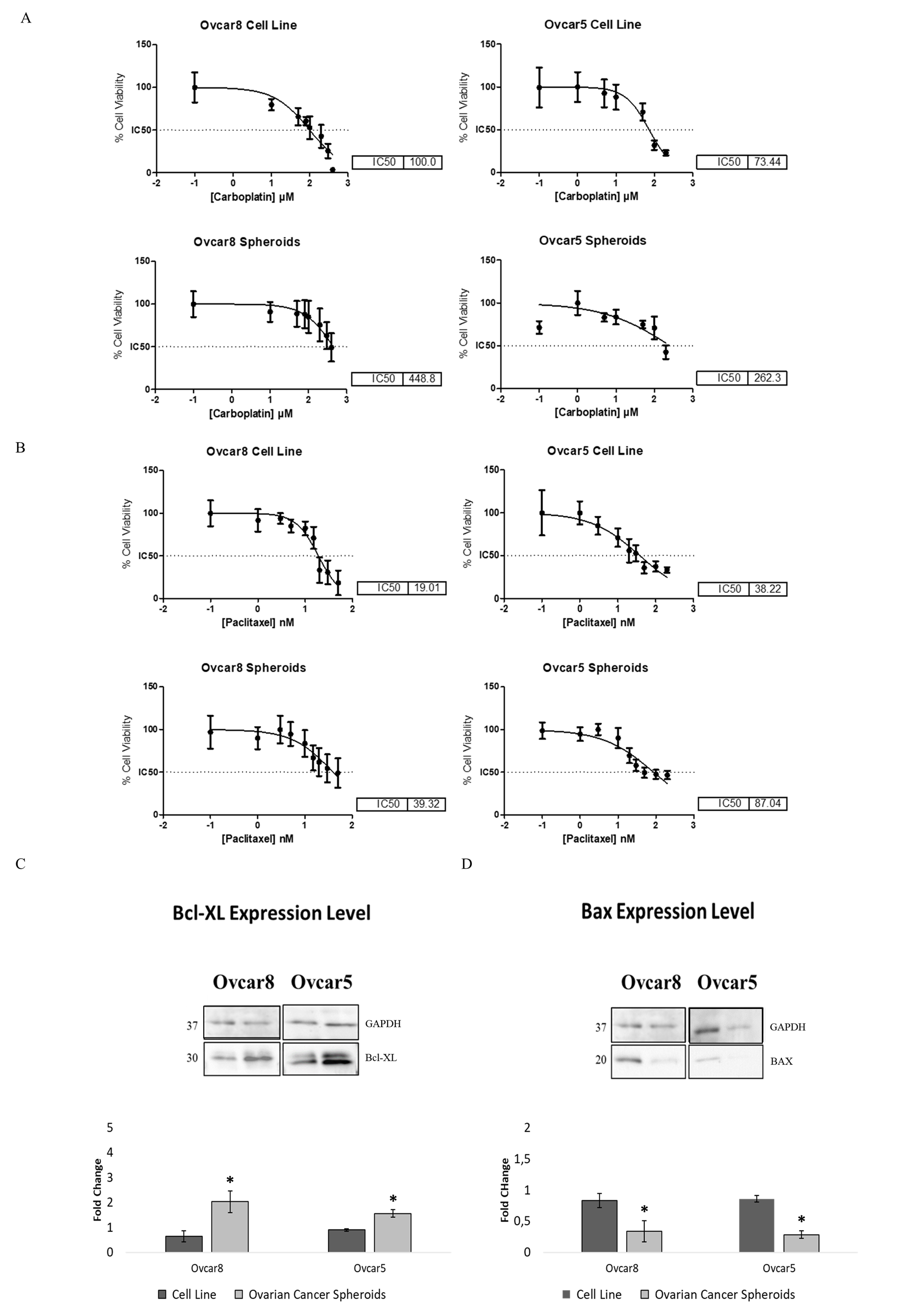

Supplement: Supplementary file 1 [file cancers-17-01605-s001.zip › Figure S1.tif]

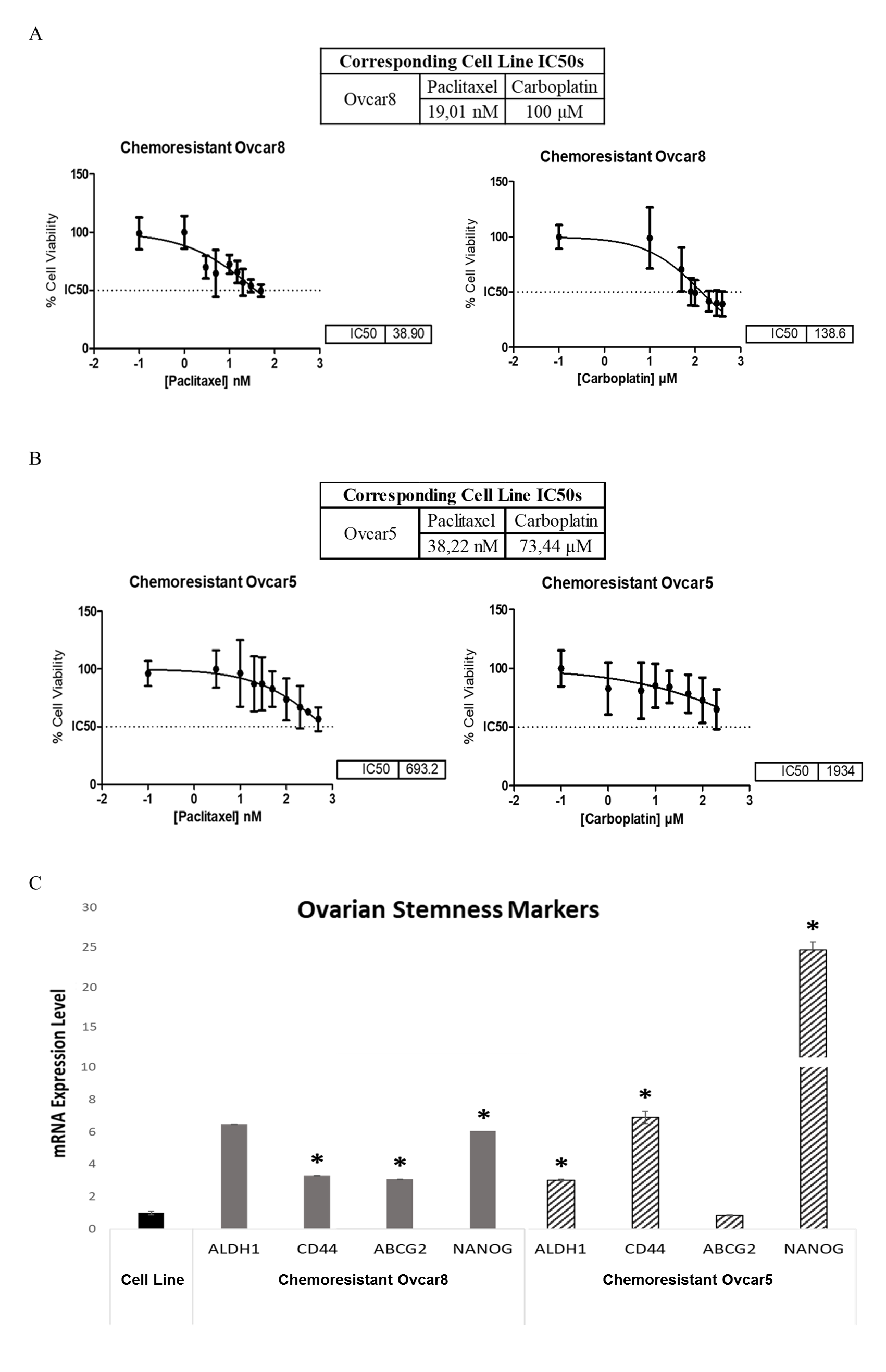

Supplement: Supplementary file 1 [file cancers-17-01605-s001.zip › Figure S2.tif]

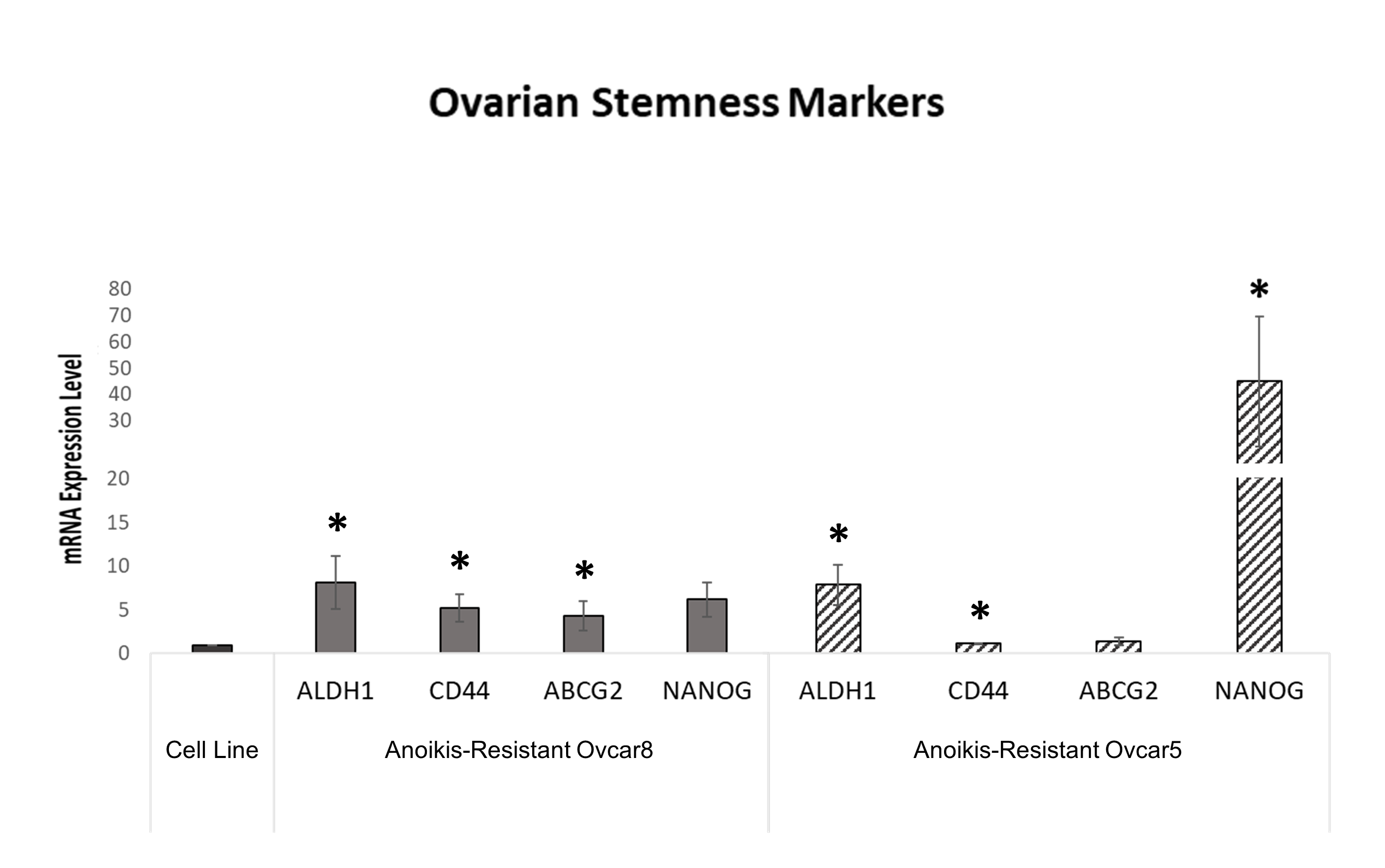

Supplement: Supplementary file 1 [file cancers-17-01605-s001.zip › Figure S3.tif]

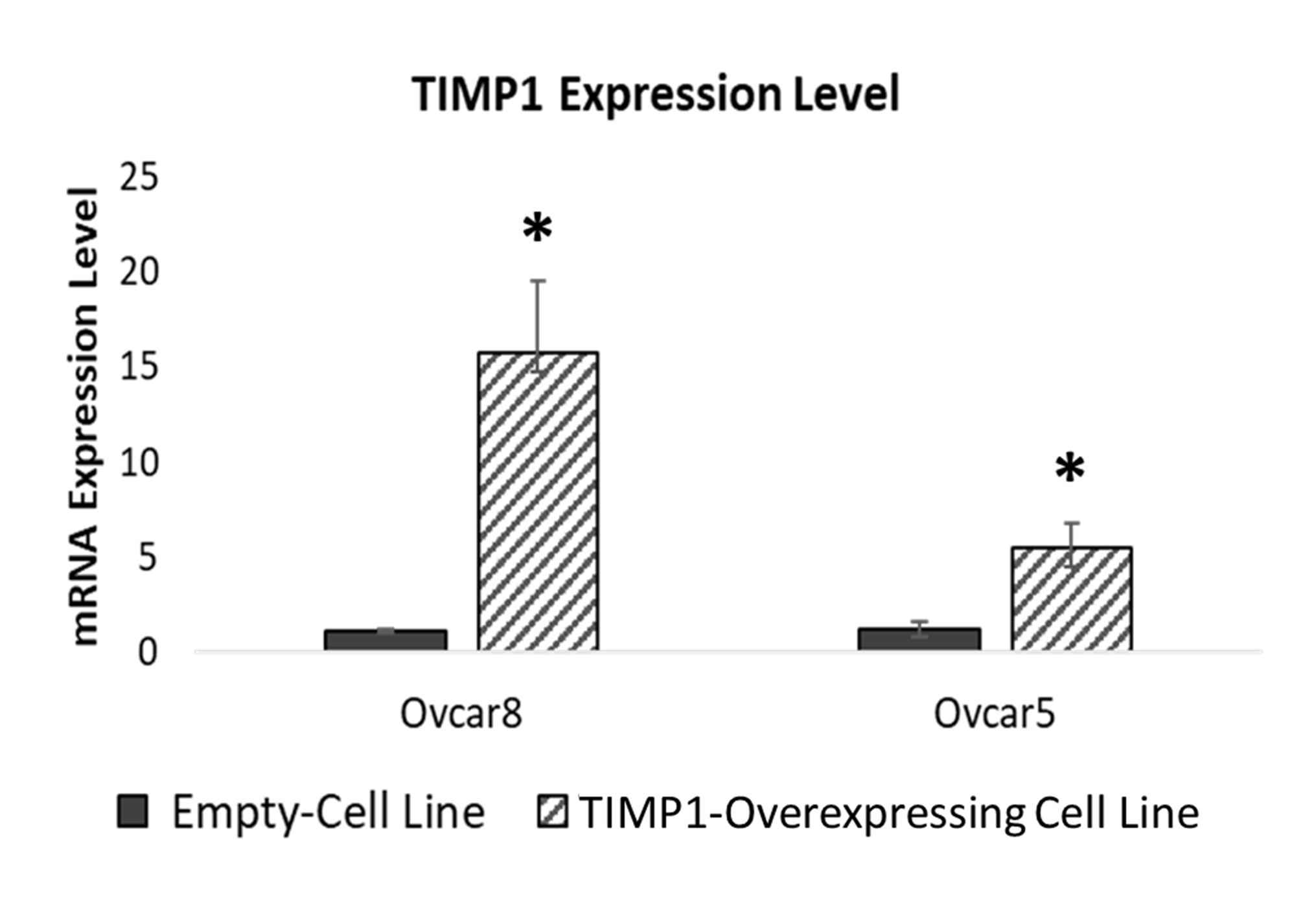

Supplement: Supplementary file 1 [file cancers-17-01605-s001.zip › Figure S4.tif]
